# Serological Measures of Trachoma Transmission Intensity

**DOI:** 10.1038/srep18532

**Published:** 2015-12-21

**Authors:** Diana L. Martin, Ryan Wiegand, Brook Goodhew, Patrick Lammie, Carolyn M. Black, Sheila West, Charlotte A. Gaydos, Laura Dize, Harran Mkocha, Mabula Kasubi, Manoj Gambhir

**Affiliations:** 1 Division of Parasitic Diseases and Malaria, Centers for Disease Control and Prevention, Atlanta GA 30329 USA; 2 National Center for Emerging, Zoonotic, and Infectious Diseases, Centers for Disease Control and Prevention, Atlanta GA 30329 USA; 3 Wilmer Eye Institute, Johns Hopkins University, Baltimore, Maryland, 21055 USA; 4 Sexually Transmitted Infections Research Laboratory, Johns Hopkins University, School of Medicine, Baltimore, MD 21205; 5 Kongwa Trachoma Project, Kongwa, Tanzania; 6 Muhimbili University, Dar es Salaam, Tanzania; 7 Epidemiological Modelling Unit, Monash University, Melbourne Australia

## Abstract

Ocular infection with *Chlamydia trachomatis* can lead to trachoma, a leading infectious cause of blindness. Trachoma is targeted for elimination by 2020. Clinical grading for ocular disease is currently used for evaluating trachoma elimination programs, but serological surveillance can be a sensitive measure of disease transmission and provide a more objective testing strategy than clinical grading. We calculated the basic reproduction number from serological data in settings with high, medium, and low disease transmission based on clinical disease. The data showed a striking relationship between age seroprevalence and clinical data, demonstrating the proof-of-principle that age seroprevalence predicts transmission rates and therefore could be used as an indicator of decreased transmission of ocular trachoma.

Trachoma is a neglected tropical disease caused by repeated ocular infection with the bacterium *Chlamydia trachomatis (Ct*). Active disease, defined as trachomatous inflammation–follicular (TF) or trachomatous inflammation–intense (TI), is self-limiting, but repeated infections can lead to pathology in the form of scarring (TS); trichiasis (TT), distinguishable by turned-in eyelashes rubbing against the globe of the eye; and irreversible blindness caused by corneal opacity (CO)^1^. Global efforts to eliminate trachoma as a public health problem by the year 2020 are based on the SAFE strategy: surgery for treatment of trichiasis, mass drug administration of antibiotics, and promotion of facial cleanliness and environmental improvement.

As elimination efforts proceed, defining programmatic endpoints becomes a priority. We have recently begun examining the utility of serological tests for post-endemic surveillance of trachoma elimination programs^2^. Antibody responses to the *Ct* antigens pgp3 and CT694 show high sensitivity and specificity for *Ct* infection^2^. A large percentage of children living in trachoma-endemic communities have detectable antibody responses but exhibit no clinical signs and lack bacterial nucleic acid in the conjunctiva, suggesting that these responses are indicators of historical rather than active infection^2^. No seroreversion was observed in seropositive individuals examined six months after drug treatment, although statistically significant decreases in antibody levels to pgp3 and CT694 were observed in younger age groups^3^.

Post-endemic testing has shown very low seropositivity in young children in the absence of infection^4^ (West *et al.*, submitted). Program end-points are currently set at <5% TF in 1–9 year-olds, operating under the assumption that low enough levels of ocular trachoma transmission will not result in downstream blindness. Since MDA will not uniformly result in zero transmission in districts, serosurveillance will only be useful to programs if there is a clear understanding of how seroprevalence relates to currently used indicators of trachoma. To this end, we evaluated age seroprevalence in communities described as hyperendemic, mesoendemic, or hypoendemic for trachoma based on TF and rates of ocular infection, and estimated the basic reproduction number (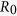) for each scenario.

## Methods

### Study Population

Studies were conducted in the Kongwa District of Tanzania as part of two separate studies in collaboration with the Kongwa Trachoma Project. The first is an ongoing study to evaluate the impact of alternative models of community-wide treatment with azithromycin and to compare nucleic acid amplification test methods^5^,^6^,^7^ (hyperendemic community). The second is a study to evaluate the health impact of an integrated NTD program on non-targeted diseases (mesoendemic and hypoendemic communities). Children 1–6 years of age were recruited from a single hyperendemic community and children 1–9 years of age were recruited from eight villages each to comprise the mesoendemic and hypoendemic communities. Clinical examinations for TF were performed by experienced graders, and dried blood spots (DBS) and conjunctival eye swabs for PCR were collected.

### Ethics Statement

Parents or guardians provided written informed consent for children participating in the study. Children over 7 provided verbal assent. The study was approved by The Institutional Review Boards of the Tanzanian National Institute for Medical Research (Dar es Salaam, TZ), Centers for Disease Control and Prevention (Atlanta, GA) for both studies and the Johns Hopkins University School of Medicine (Baltimore, MD) for the first study (hyperendemic community) only. The study was carried out in accordance with the approved guidelines.

### Grading of Ocular Trachoma

Clinical exams, using the WHO simplified grading scheme^8^, were performed on children aged 1 to 9 years from nine villages in Kongwa District by an experienced trachoma grader. TF was graded as negative if the ocular signs did not meet WHO criteria of positive TF with 5 or more follicles of greater than 0.5 mm^8^.

### Nucleic Acid Amplification Testing

Eye swabs were collected for PCR analyses of *C. trachomatis* from all children, with careful attention to avoid field contamination. Swabs from the hyperendemic community were sent to the International Chlamydia Research Laboratory at Johns Hopkins University and tested for the presence of chlamydial DNA using Amplicor CT/NG (Roche, Basel, Switzerland) as described^3^,^6^. According to the manufacturer’s directions, the Amplicor test was positive if the optical density read at 450 nM was >0.8, negative if the signal was <0.2, and equivocal if in-between. All equivocal tests were re-tested in duplicate, and only graded positive if at least one test was positive. PCR testing in hypo- and meso-endemic settings was done at CDC on non-pooled ocular swabs using a nested PCR protocol (Expand High Fidelity PCR System, Roche Diagnostics Corporation, Indianapolis, IN) to amplify the *ompA* gene as previously described^9^.

### Serology for Assessment of chlamydial-specific Antibodies

Serum was eluted from dried blood spots and then incubated with chemically-modified microspheres (Luminex Corp., Austin, TX) conjugated to the *Ct* antigens pgp3 and CT694^2^. After washing out unbound serum antibodies, bound antibody was detected with biotinylated mouse anti-human IgG (clone H2; Southern Biotech, Birmingham, AL) and biotinylated mouse anti-human IgG_4_ (clone HP6025; Invitrogen, South San Francisco, CA), followed by *R*-phycoerythrin-labeled streptavidin (SAPE, Invitrogen, South San Francisco, CA). Beads were suspended in 125 μl PBS, shaken, and immediately read on a BioPlex 200 instrument (Bio-Rad, Hercules, CA) equipped with Bio-Plex Manager 6.0 software (Bio-Rad).

### Modeling

Because it is not possible to directly measure the transmission contact pattern over age groups, the basic reproduction rate (*R*_0_) was calculated to estimate the transmission potential using a simple method that assumes a constant force of infection (cFOI) over age^10^. While this is a crude approximation of the true contact pattern, which is likely to differ over age, the resulting estimate of *R*_0_ serves as a simple summary parameter, allowing quick comparison of each community. In addition, by using this very simple approach, we can compare the data in this study with serological data from other studies. A close correspondence between estimates obtained by the cFOI method and those from the more complex ‘Next Generation Matrix’ (NGM) method^11^ for age-dependent models fitted to serological data have been observed in a recent model of cytomegalovirus infection, which has, if anything, a more complex transmission pattern and natural history than trachoma^10^. Furthermore, because the serological data analyzed here pertain primarily to young children, the assumptions of the cFOI model might be expected to be roughly true; they should, however, be restricted to young children and not the whole population. The proportion of the population at age *a* that is seronegative 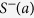 (with force of infection at age *x*, denoted by λ(*x*)) is given by:


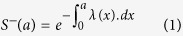


Assuming that the force of infection is constant with age we see that:


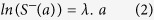


Lanzieri *et al.*^1010109^ state that, in this case, *R*_*0*_ can be approximated as:


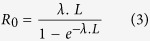


where *L* is the average lifespan of the population, which we set to 75 years here. This value of the lifespan may not correspond exactly with the true average life expectancy across the regions from which data were collected. However, the value of *L* is used here to set the scale of the calculated *R*_0_; since this *R*_0_ does not pertain to the whole population it should simply be set to a fixed value to allow comparison of its value across different countries. The values of 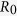 obtained were approximately 15% smaller if a value of *L* equal to 63 years (Tanzanian value http://data.worldbank.org/indicator/SP.DYN.LE00.FE.IN) was used. Confidence interval (CI) 95% bounds were reported by inserting the 95% upper and lower bound values for the force of infection 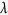 into the formula for 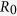 above. The values of 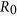 obtained by the cFOI method were checked against those calculated for hyper-, meso- and hypoendemic communities using the NGM method from a previous mathematical modeling study^12^. The NGM method used here assumes an initially naive susceptible population, which has experienced minimal prior infections; as such, the NGM method can also be said to apply to young children and hence correspond to the cFOI method used here for the same age group. All calculations were performed using Matlab version R2013b (Mathworks, MA).

## Results

**Figure 1:**
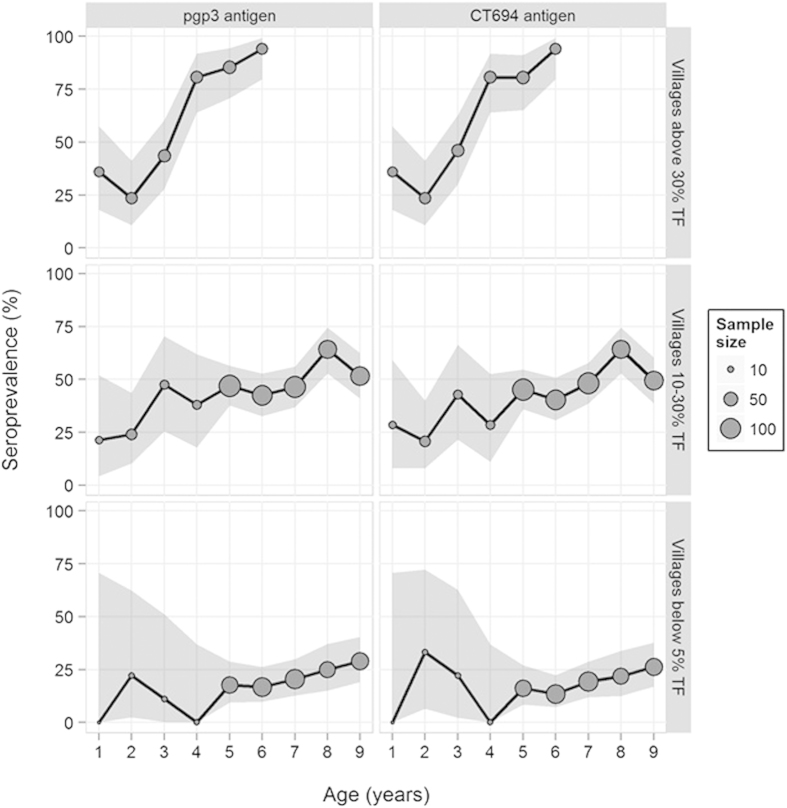
Seroprevalence percentages by age broken down by antigen (pgp3 and CT694) and village prevalence (below 5% TF, 10-30% TF, and above 30% TF). Seroprevalences are represented with circles scaled by the sample size and shaded regions denote 95% confidence intervals. For antigen-specific seroprevalences by age, confidence intervals use the incomplete beta function^19^ to account for village-level clustering (except when the seroprevalence was zero and Clopper-Pearson (1934) limit^19^ were used).

In a hyperendemic setting, 208 1–6 year-old children from a single village in Kongwa District, TZ, had a TF prevalence of 47% and ocular infection rates of 24% (previously published in^3^,^6^, Table 1). In the mesoendemic villages, TF prevalence in 987 1-9 year olds was 14·5% and ocular infection rate was 8·3% (Table 1). For the hypoendemic setting, TF was present in 2·9% and ocular infection in 1·8% of 680 1–9 year-olds evaluated (Table 1). Antibody levels against the *Ct* antigens pgp3 and CT694 were determined for 208 children in the hyperendemic setting, 581 children in the mesoendemic communities, and 419 children in the hypoendemic communites. The data from the four mesoendemic villages and four hypoendemic villages were aggregated into the two sample sets for analysis. TF, NAAT, and serology data are shown in Table 1. Figure 1 shows age-seroprevalence curves, with 95% CIs in the shaded area.

Using the cFOI method, we estimated the value of *R*_0_ in young children for each setting using age seroprevalence against each antigen (pgp3 and CT694; Table 1). 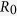 (pgp3) was 29·4 for the hyperendemic community, 8·1 for the mesoendemic setting, and 2·8 for the hypoendemic setting. 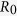 (CT694) was 28·3 for the hyperendemic community, 7·8 for the mesoendemic setting, and 2·8 for the hypoendemic setting (95% confidence intervals for these values are provided in Table 1). These values of the basic reproduction number correspond well with equivalent values in young children calculated for hyper-, meso- and hypoendemic communities using the NGM method for a more complex epidemiological model fitted to baseline infection prevalence data over all age groups in the community^12^: hyperendemic 34·9 [27.5, 44.2] (95% CIs, based upon the transmission rate uncertainty), mesoendemic 3·0 [2.5, 3.6], hypoendemic 2·3 [2.0, 2.7]. It should be noted that the meso- and hypoendemic study sites whose data were used in the NGM calculation differed from the Kongwa District, Tanzania, study site used for the serological calculations.

## Discussion

Our data demonstrate that age seroprevalence provides a proxy for clinical disease in determining transmission rates for trachoma. A conspicuous increase in the slope of the age seroprevalence curve was seen with increasing TF prevalence in the setting, which was reflected in the numerical value of 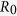 for each setting. Modelling showed clear differences in the seroprevalence-determined 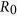 among young children in the hyperendemic, mesoendemic, and hypoendemic settings, regardless of the antigen used.

The 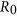 values obtained here are higher than those previously described using model-based estimates^13^,^14^, although those studies estimated 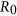 over the whole population, with all age groups combined, using a stochastic transmission model used to fit cross-sectional ocular infection data. This would be expected to result in a substantially different 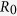 estimate because the rate of recovery from infection is thought to increase and the bacterial load per infection to decrease with each subsequent infection, and therefore with age^15^,^16^,^17^,^18^. Contact rates are also likely to drop in older age groups, and these effects all lead to lower forces of infection and contributions to the overall reproduction number. We emphasize that we are calculating an 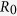 here that applies to the age groups surveyed i.e. young children.

The major limitations of this study are the relatively small sample size, particularly the small number of 1–4 year olds in the mesoendemic and hypoendemic settings, and the lack of 7–9 year olds in the high-prevalence village. This is especially apparent in the wide confidence intervals for antibody responses in 1–4 year olds in the hypo and mesoendemic communities as shown in Fig. 1. We would predict that since 92% of 6-year-olds in the high prevalence village were seropositive that this trend would continue with the older ages but still need to generate the data to support that. Having the 7–9 year-olds in that village may also have changed the 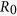 calculated by the model, although if the seroprevalence trend continued at >90% in 7–9 year-olds in that village, the *R*_0_ would almost certainly be greater than the current estimate. Changes in antibody levels after treatment have only been examined in a single high prevalence community^3^, and changes over time in the absence of treatment have not been evaluated at all. Therefore assumptions about negligible seroreversion in all settings may be premature. Currently, studies are underway examining 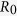 and force of infection in hyperendemic, mesoendemic, and hypoendemic-prevalence settings at the district level, the implementation and evaluation units for trachoma programs. We will also evaluate changes in antibody levels in a variety of transmission settings, as the high attack rate in hyperendemic communities may result in earlier formation of long-lived antibody-producing plasma cells than in areas with lower attack rates.

Despite the limitations, the data provide a powerful proof-of-concept that age seroprevalence predicts transmission rates of trachoma and therefore could be used as an indicator of decreased exposure. Post-endemic surveillance is a difficult sell to donors and to programs, so creative approaches to surveillance are urgently needed. This is especially true for trachoma programs, for which decision-making is guided by personnel and training-intensive ocular examinations. A serosurveillance platform has the potential for integration with other surveillance activities, making the most of the increasingly limited resources available to elimination programs.

## Additional Information

**How to cite this article**: Martin, D. L. *et al.* Serological Measures of Trachoma Transmission Intensity. *Sci. Rep.* **5**, 18532; doi: 10.1038/srep18532 (2015).

## Figures and Tables

**Table 1:** Overall prevalence of clinical signs, ocular infection, antibody-positivity, and calculated 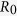 in hyperendemic, mesoendemic, and hypoendemic settings.

	Age range	%TF+ [95% CI)	%NAAT + [95% CI]	% pgp3Ab + (95% CI)	%CT694 Ab+ [95% CI]	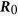 pgp3 [95% CI]	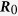 CT694 [95% CI]
High	1–6	47.0 [44.7–49.3]	24·0 [17.2–30.8]	62·0 [55.4–68.6]	61·5 [54.9–68.1]	29·4 [21·1–37·7]	28·3 [19·9–36·8]
Medium	1–9	14·5 [12.3–16.7]	8·1 [6.4–9.8]	3·0 [30.0–36.0]	34·0 [31.0–37.0]	8·1 [6·3–10·0]	7·8 [6·0–9·6]
Low	1–9	2·8 [1.4–4.2]	2·0 [0.82–3.2]	21·2 [17.7–24.6]	18·4 [15.1–21.7]	2·8 [2·1–3·6]	2·8 [1·6–4·0]
